# Inhibition of immunoproteasome reduces infarction volume and attenuates inflammatory reaction in a rat model of ischemic stroke

**DOI:** 10.1038/cddis.2014.586

**Published:** 2015-01-29

**Authors:** X Chen, X Zhang, Y Wang, H Lei, H Su, J Zeng, Z Pei, R Huang

**Affiliations:** 1 Department of Neurology, National Key Clinical Department and Key Discipline of Neurology, Guangdong Key Laboratory for Diagnosis and Treatment of Major Neurological Diseases, The First Affiliated Hospital, Sun Yat-Sen University, Guangzhou 510080, PR China; 2 Department of Neurology, Fujian Provincial Hospital, Fujian Medical University Shengli Clinical College, Fuzhou 350001, PR China; 3 Key Laboratory of Quality Research in Chinese Medicine, Institute of Chinese Medical Sciences, University of Macau, Macao, PR China

## Abstract

The detailed knowledge about the contribution of immunoproteasome to the neuroinflammation in ischemic stroke is still not available. The immunoreactivity of low molecular mass peptide 2 (LMP2) and low molecular mass peptide 7 (LMP7) was evident in the ipsilateral ischemic cerebral cortex and striatum following transient middle cerebral artery occlusion (MCAO). Both LMP2 and LMP7 increased as early as 4 h after the MCAO, further increased at 24 h, peaked at 72 h and decreased 7 days later. LMP2 and LMP7 were mainly present in astrocytes and microglia/macrophage cells, respectively. LMP2 knockdown by shRNA (short hairpin RNA) markedly reduced the levels of LMP2 and LMP7 protein and caused 75.5 and 78.6% decrease in the caspase-like (C-L) and chymotrypsin-like (CT-L) activities, respectively. Compared with cont-shRNA group (39.7%, infarction volumes/total ipsilateral hemisphere), the infarction volumes were reduced to 22.5% in LMP2-shRNA group. Additionally, LMP2 knockdown significantly reduced activated astrocytes and microglia, the expression nuclear factor kappa B (NF-*κ*B) p65, tumor necrosis factor-*α* (TNF-*α*) and interleukin-1*β* (IL-1*β*) and caused less accumulation of ischemia-induced protein ubiquitination compared with MG132. These findings demonstrate that inhibition of LMP2 significantly attenuates inflammatory reaction and offers neuroprotection against focal cerebral ischemia in rats, suggesting that selective immunoproteasome inhibitors may be a promising strategy for stroke treatment.

A wealth of data from both human and animal studies indicates that inflammation has a pivotal role in ischemic injury.^1^ It is generally believed that excessive inflammation mediates ischemic injury whereas blockade of inappropriate inflammatory responses improves ischemic injury.^1^ Normally, inflammatory processes are heavily controlled by complex endogenous regulatory systems such as proteasome system. The proteasome system is the major system for intracellular protein degradation and regulates inflammatory processes by degradation of inflammation-associated molecules including NF-*κ*B, a critical transcription factor for numerous proinflammatory genes.^2^ Proteasome has been reported to activate NF-*κ*B through degradation of the inhibitory subunit of NF-*κ*B, which in turn induces inflammatory processes.^3^ In contrast, proteasome inhibitors exhibit anti-inflammatory actions in a variety of inflammatory-related diseases. In the field of stroke, several general proteasome inhibitors such as MLN519 have been demonstrated to reduce post-stroke inflammatory and protect against cerebral ischemia.^4, 5^ However, the clinical uses of general proteasome inhibitors are often limited by their side effects due to their non-selective inhibition of protein degradation.^6^ Thus, selectively targeting proteasome elements responsible for inflammatory pathways would increase efficacy and reduce side effects.

Immunoproteasome is a subtype of proteasome, which is predominantly present in the immune cells. Immunoproteasome contains two major catalytic subunits: LMP2 (low-molecular weight protein 2, PSMB9, *β*1i) and LMP7 (low-molecular weight protein 7, PSMB8, *β*5i). It has been demonstrated that immunoproteasome has a critical role in regulating the production of proinflammatory cytokines^7^ and maintaining protein homeostasis under cytokine-induced oxidative stress by preventing protein aggregate formation.^8, 9^ Blockage of LMP7 with immunoproteasome-specific inhibitor PR957 affords a strongly attenuation of disease and a suppression of proinflammatory cytokines such as TNF-*α*, IL-23 and IL-2.^7, 10^

Immunoproteasome and its subunits have been linked to a variety of human diseases including central nervous system (CNS) diseases such as Alzheimer's (AD)^11^ and Huntington's diseases (HD).^2, 12^ In 2006, Mishto *et al.*^11^ first reported the presence and role of immunoproteasome and its LMP2 subunit polymorphism at codon 60 in AD. In their study, immunoproteasome is present in neurons, astrocytes and endothelial cells in the brain areas such as the hippocampus and the cerebellum. In addition, expression of immunoproteasome is higher in the brain of AD patients than in the brain of non-demented elderly. Recently, upregulated immunoproteasomal subunits have been also observed in a mouse model of cerebral ischemia,^13^ suggesting the involvement of immunoproteasome in ischemic stroke. However, the function of immunoproteasome in cerebral ischemia remains elusive. In the present study, we investigated the time course and cellular distribution and function of immunoproteasome subunit LMP2 and LMP7 in the rat brain following cerebral ischemia-reperfusion.

## Results

### Expression and cellular localization of LMP2 in the brains of MCAO rats

**Figure 1:**
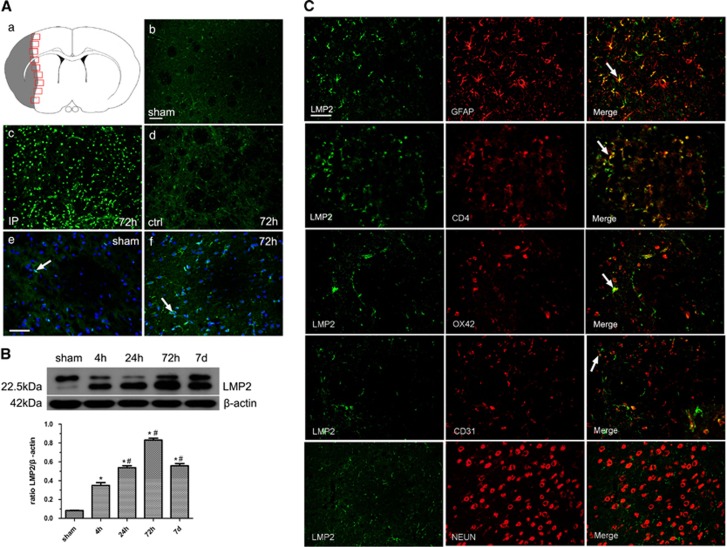
Expression and cellular localization of LMP2 in the brains of MCAO rats. (**A**) A schematic representation of a coronal brain section. The square fields represent observed regions (a). There was only sparse weakest immunoreactivity of LMP2 in glia-like cells in the cortex and striatum in non-ischemic hemisphere and sham-operated control rats (b). In contrast, the number of cells expressing strong LMP2 immunoreactivity was significantly higher in the ipsilateral ischemic hemisphere than in the contralateral hemisphere at different time points (c and d, 72 h time point was shown here). LMP2 was mostly localized in cell nucleus in non-ischemic hemisphere whereas LMP2 was evident in both the nucleus and the cytoplasm after MCA occlusion (arrow) (e and f). Five rats in each group were used for immunofluorescent assays (*n*=5). (**B**) Western blot showed that LMP2 protein levels were significantly elevated in the ipsilateral cerebral cortex and striatum all time points when compared with sham-operated control animals. Quantification analysis confirmed that the level of LMP2 protein changed from 4 h to 7 days in the ipsilateral ischemic cortex and striatum (*P<0.001, compared with the controls; ^#^*P*<0.001 compared with the previous time point) (**B**). Five rats in each group were used for western blot (*n*=5). (**C**) Double immunostaining showed that the overwhelming majority of LMP2 (green) was colocalized with glial fibrillary acid protein (GFAP)-positive astrocytes (red) although some LMP2-positive cells were colocalized with CD4^+^T lymphocytes (red), with OX42-positive microglia/macrophage cells (red) and with CD31-positive vascular endothelial cell (red) (arrow). In contrast, LMP2-positive cells were not colocalized with NEUN-positive neurons (red) at any time points (figure 1C). Scale bars=80 *μ*m (in **A**: b–d), 50 *μ*m (in **A**: e and f, **C**). IP, ipsilateral hemisphere; ctrl, contralateral hemisphere; sham, sham-operated control rats

We first examined immunoproteasome in the brains of rats subjected to 1 h of ischemia followed by 4 h, 24 h, 72 h and 7 days of reperfusion. There was only sparse immunoreactivity of LMP2 in glia-like cells in the cortex and striatum in non-ischemic hemisphere and sham-operated control rats (Figure 1Ab). In contrast, the number of cells expressing strong LMP2 immunoreactivity was significantly higher in the ipsilateral ischemic hemisphere than in the contralateral hemisphere at different time points (Figure 1Ac−d). LMP2 immunoreactive cells were predominately localized in the penumbra surrounding the infarct core (Figure 1Ac−d). Furthermore, LMP2 was mostly localized in cell nucleus in non-ischemic hemisphere whereas LMP2 was evident in both the nucleus and the cytoplasm after MCA occlusion (Figure 1Ae−f). Western blot showed that LMP2 changed in a time-dependent manner, namely, LMP2 increased as early as 4 h after reperfusion further increased at 24 h, peaked at 72 h and decreased at 7 days. LMP2 protein levels were significantly elevated in the ipsilateral cerebral cortex and striatum at all time points when compared with sham-operated control animals. Quantification analysis confirmed that the level of LMP2 protein changed from 4 h to 7 days in the ipsilateral ischemic cortex and striatum (*P<0.001, compared with the controls; ^#^*P*<0.001 compared with the previous time point) (Figure 1B).

We further determined the cell type(s) expressing LMP2 following ischemia using double immunostaining with antibodies against cell-specific antigens. The results showed that the overwhelming majority of LMP2 (green) was colocalized with glial fibrillary acid protein (GFAP)-positive astrocytes (red) although some LMP2-positive cells were colocalized with CD4^+^T lymphocytes (red), with OX42-positive microglia/macrophage cells (red) and with CD31-positive vascular endothelial cell (red). In contrast, LMP2-positive cells were not colocalized with NEUN-positive neurons (red) at any time points (Figure 1C).

### Expression and cellular localization of LMP7 in the brains of MCAO rats

**Figure 2:**
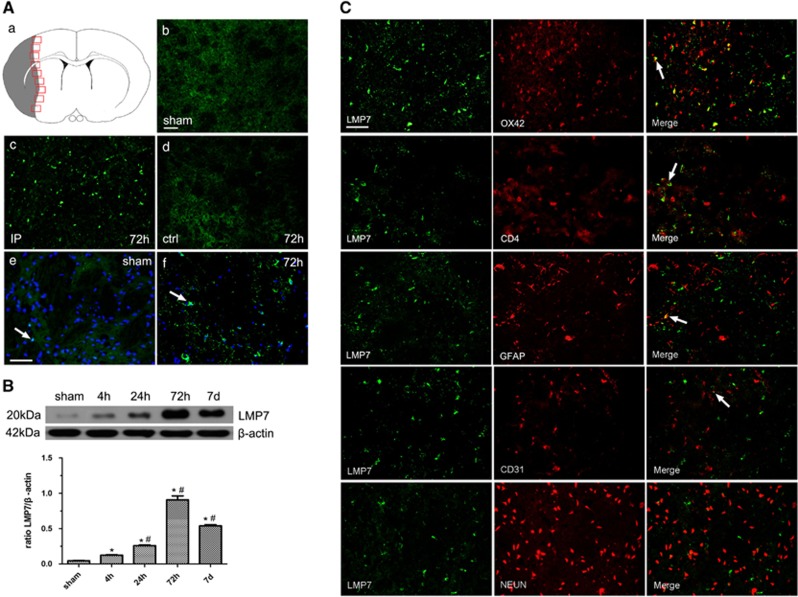
Expression and cellular localization of LMP7 in the brains of MCAO rats. (**A**) A schematic representation of a coronal brain section. The square fields represent observed regions (a). LMP7 signal was barely detectable in the cortex and striatum in the sham-operated animals and contralateral hemisphere in ischemic rats (b). In contrast, the LMP7 signal was evident in the ipsilateral ischemic hemisphere (c and d, 72 h time point was shown here). LMP7 was mostly present in the nucleus in non-ischemic hemisphere whereas it was evident in both the nucleus and the cytoplasm in ischemia hemisphere (arrow) (e and f). Five rats in each group were used for immunofluorescent assays (*n*=5). (**B**) Western blot confirmed that LMP7 protein levels were significantly elevated in the ipsilateral cerebral cortex and striatum at all times when compared with sham-operated control animals. Quantification analysis confirmed that the level of LMP7 protein changed from 4 h to 7 days in the ipsilateral ischemic cortex and striatum (*P<0.001, compared with the sham-operated controls; ^#^*P*<0.001 compared with the previous time point) (**B**). Five rats in each group were used for western blot (*n*=5). (**C**) Double immunostaining indicated that LMP7 was mainly expressed in OX42-positive microglia/macrophage cells (red), some in CD4^+^T lymphocytes (red) and very few LMP7 in glial fibrillary acid protein (GFAP)-positive astrocytes (red) as well as CD31-positive vascular endothelial cell (red) (arrow). In contrast, LMP7 was not detected in NEUN-positive neurons (red) at any time points (**C**). Scale bars=80 *μ*m (in **A**: b–d), 50 *μ*m (in **A**: e and f, **C**). IP, ipsilateral hemisphere; ctrl, contralateral hemisphere; sham, sham-operated control rats

The immunofluorescent analysis of rat brain sections showed that LMP7 signal was barely detectable in the cortex and striatum in the sham-operated animals and contralateral hemisphere in ischemic rats (Figure 2Ab). In contrast, the LMP7 signal was evident in the ipsilateral ischemic hemisphere (Figure 2Ae−f). LMP7 was mostly present in the nucleus in non-ischemic hemisphere whereas it was evident in both the nucleus and the cytoplasm in ischemia hemisphere (Figure 2Ac−d). Similar to LMP2, LMP7 changed significantly from 4 h to 7 days in response to ischemia insult. Western blot confirmed that LMP7 protein levels were elevated after 4 h of cerebral ischemia/reperfusion, followed by a moderate further increase at 24 h and peaked at 72 h, whereas the intensity tended to decrease at 7 days. Quantification analysis confirmed that the level of LMP7 protein changed from 4 h to 7 days in the ipsilateral ischemic cortex and striatum (*P<0.001, compared with the sham-operated controls; ^#^*P*<0.001 compared with the previous time point) (Figure 2Ae−f).

To investigate the cell type(s) expressing immunoproteasome LMP7, brain sections were double-labeled using antibodies against cell-specific antigens. LMP7 was mainly expressed in OX42-positive microglia/macrophage cells (red), some in CD4^+^T lymphocytes (red) and very few LMP7 in GFAP-positive astrocytes (red) as well as CD31-positive vascular endothelial cell (red). In contrast, LMP7 was not detected in NEUN-positive neurons (red) at any time points (Figure 2C). Thus, our findings indicate that immune cells are the major producers for LMP7.

### Expression of *β*1, *β*5 and 20S a subunits in the brains of MCAO rats

**Figure 3:**
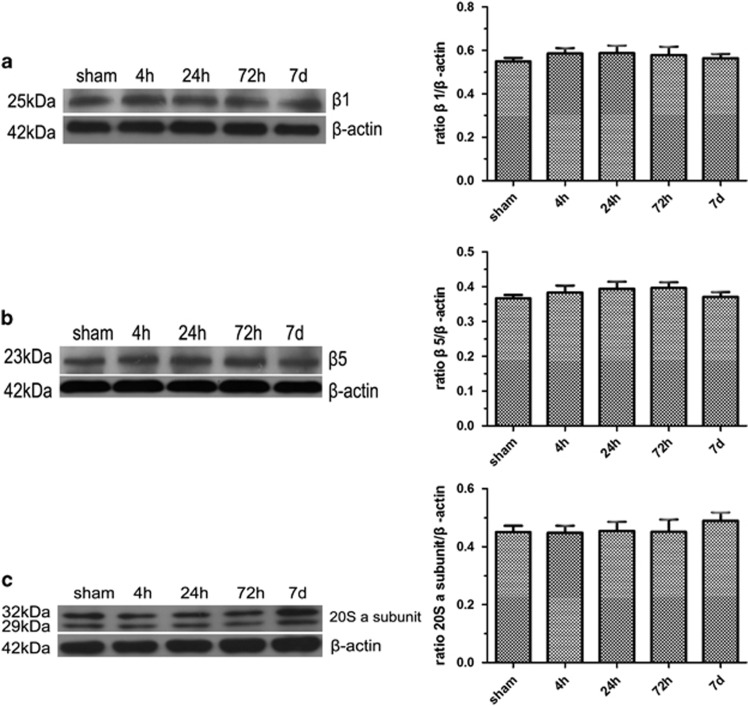
Expression of *β*1, *β*5 and 20S *α* subunits in the brains of MCAO rats. Western blot confirmed that protein levels of *β*1, *β*5 and 20S *α* subunits (1,2,3,5,6,7) did not show a significant change from 4 h to 7 days in the cortex and striatum under ischemia condition, respectively (*P*>0.10, compared with the sham-operated group; *P*>0.10 compared with the previous time point) (**a**–**c**). Five rats in each group were used for western blot (*n*=5), sham, sham-operated control rats

Meanwhile, *β*1, *β*5 standard subunit as well as a constitutive proteasome subunit of the 20S proteasome *α* subunits (1,2,3,5,6,7) did not show a significant change in the same brain region examined under the same conditions, respectively (*P*>0.10, compared with the sham-operated group; *P*>0.10 compared with the previous time point) (Figure 3A−C).

### Expression of TNF-*α*, IL-1*β* and NF-*κ*B in the brains of MCAO rats

**Figure 4:**
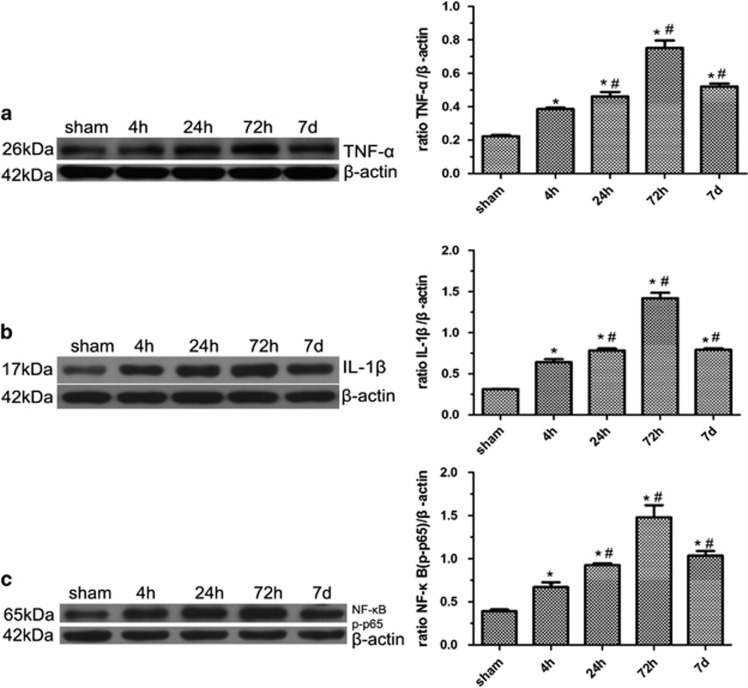
Expression of TNF-*α*, IL-1*β* and NF-*κ*B in the brains of MCAO rats. Western blot confirmed that protein levels of TNF-*α*, IL-1*β* and phospho-NF-*κ*B p65(NF-*κ*B p-p65) increased as early as 4 h after reperfusion, further increased at 24 h, peaked at 72 h and decreased at 7 days, respectively (*P<0.001, compared with the controls; ^#^*P*<0.001 compared with the previous time point) (**a**–**c**). Five rats in each group were used for western blot (*n*=5), sham, sham-operated control rats

To investigate the potential link between inflammation and immunoproteasome, we examined the expression of inflammatory cytokines such as TNF-*α*, IL-1*β* and NF-*κ*B. Consistent with the time course of immunoproteasome expression, TNF-*α*, IL-1*β* and Phospho-NF-*κ*B p65(NF-*κ*B *P*-p65) increased as early as 4 h after reperfusion, further increased at 24 h, peaked at 72 h and decreased at 7 days (*P<0.001, compared with the controls; ^#^*P*<0.001 compared with the previous time point) (Figure 4A−C). Correlation analysis revealed a significant association of immunoproteasome with TNF-*α*, IL-1*β* and NF-*κ*B p65.

### LMP2 knockdown by shRNA and immunoproteasome expression as well as proteasome activity in the brain after MCAO

**Figure 5:**
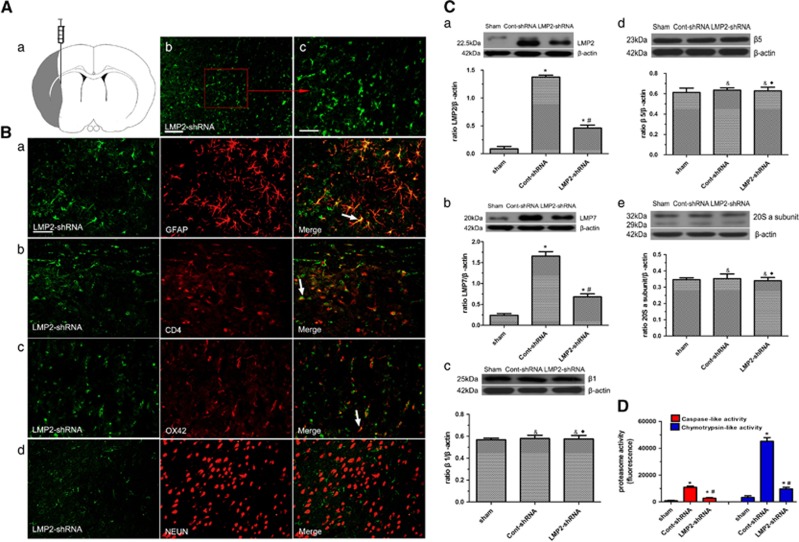
LMP2 knockdown by shRNA and immunoproteasome expression as well as proteasome activity in the brain after MCAO. (**A**) Schematic diagram of brain section showing the location of cortical and striatum infarction (black color) and where lentivirus was injected (a). EGFP-tagged shRNA was detected in the cortical and the striatum at 72 h after MCAO (b and c). (**B**) Immunofluorescence labeling showed that EGFP-tagged LMP2-shRNA was expressed in GFAP^+^, CD4^+^T lymphocytes and OX42 ^+^cells not in NEUN-positive neurons (arrow) (a–d). Five rats in each group were used for immunofluorescent assays (*n*=5). (**C**) Western blot results demonstrated that LMP2 knockdown markedly decreased the protein levels of LMP2 and LMP7 compared with cont-shRNA group. Five rats in each group were used for western blot (*n*=5). (**D**) MCAO induced a robust increase in C-L and CT-L activities compared with the sham-operated controls, whereas LMP2-shRNA led to decreased in C-L and CT-L activities compared with cont-shRNA group, respectively (*P<0.001, ^&^*P*>0.10, compared with the sham-operated controls; ^#^*P*<0.001, ^♦^*P*>0.10, compared with the cont-shRNA group) (figure 5). Scale bars=80 *μ*m (in **A**: b), 50 *μ*m (in **A**: c, **B**)

To determine the potential roles of LMP2 in the brain after MCAO, lentivirus-mediated LMP2 shRNA (referred to as LMP2-shRNA) or lentivirus vector carrying scrambled shRNA (referred to as cont-shRNA) was injected stereotactically into the right cortex and striatum in experimental rats. Immunofluorescence labeling was used to reveal the local injection of EGFP-tagged LMP2-shRNA into the right cortex and striatum in experimental rats (Figure 5Aa−c). Double immunostaining showed that the overwhelming majority of LMP2-shRNA (green) was colocalized with GFAP-positive astrocytes (red). Some LMP2-shRNA-positive cells were colocalized with CD4^+^T lymphocytes and with OX42-positive microglia/macrophage cells (red). In contrast, there was not colocalization between NEUN-positive neurons (red) and LMP2-shRNA positive cells at any time point (Figure 5Ba−d). Western blot results demonstrated that LMP2 knockdown markedly decreased the protein levels of LMP2 and LMP7, decreased 66.5 and 58.9% compared with cont-shRNA group, but did not affect the protein levels of *β*1, *β*5 standard subunit as well as 20S proteasome *α* subunits (1,2,3,5,6,7) (Figure 5Ba−e). We further measured the proteasome-dependent proteolytic activities (the caspase-like (C-L) and chymotrypsin-like (CT-L) activities) after MCAO. MCAO induced a robust increase in C-L and CT-L activities (about 12-fold and 13-fold increase compared with the sham-operated controls, respectively), whereas LMP2-shRNA caused nearly 75.5 and 78.6% decrease in C-L and CT-L activities compared with cont-shRNA group, respectively (*P<0.001, ^&^*P*>0.10, compared with the sham-operated controls; ^#^*P*<0.001, ^♦^*P*>0.10, compared with the cont-shRNA group) (Figure 5D).

### LMP2 knockdown by shRNA and MG132 pretreatment reduced infarction volumes in rats

**Figure 6:**
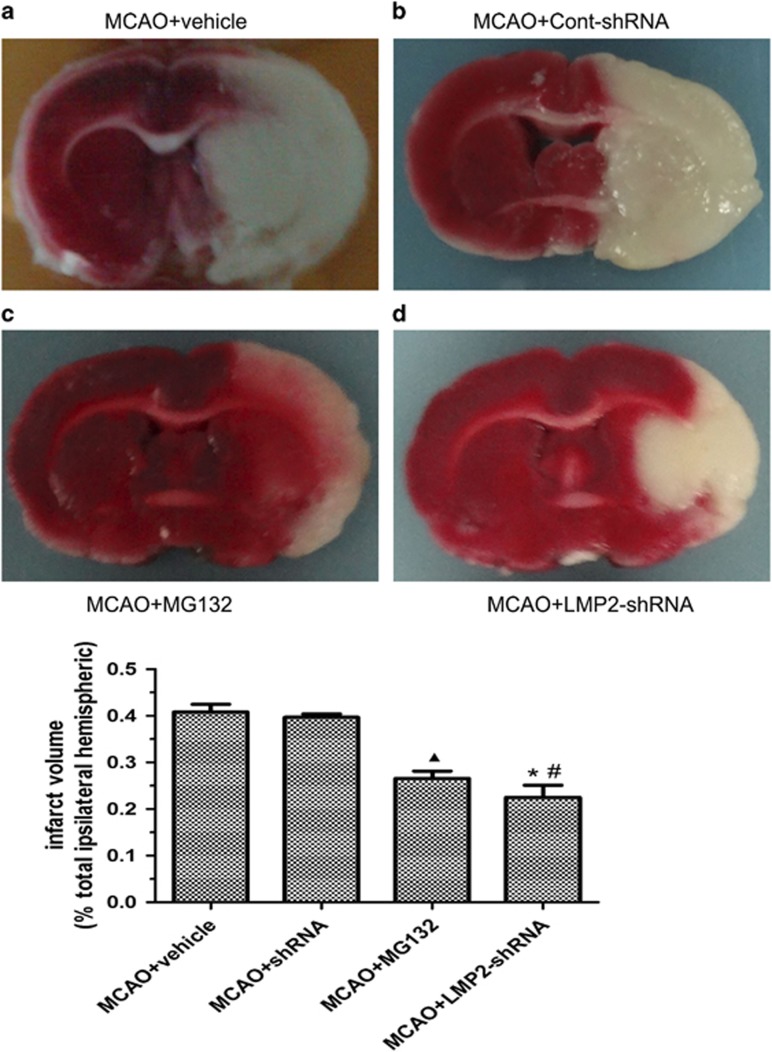
LMP2 knockdown by shRNA and MG132 pretreatment reduced infarction volumes in rats. The sections were stained by incubating them in a solution of 2% 2,3,5-triphenyltetrazolium chloride (TTC) and the infarcted brain tissue appeared white, whereas the non-infarcted region appeared red. LMP2 knockdown by shRNA significantly reduced infarction volumes in ischemic stroke in rats. Compared with MCAO+vehicle group (40.8%, infarction volumes/total ipsilateral hemispheric), the infarction volumes were reduced to 26.6% in MCAO+MG132 group (^▴^*P*<0.001, compared with MCAO+vehicle group) (**a** and **c**). Similarly, the infarction volumes were reduced to 22.5% in LMP2-shRNA group compared with Cont-shRNA group (39.7%, infarction volumes/total ipsilateral hemispheric) (*P<0.001, compared with Cont-shRNA group) (**b** and **d**).The infarction volumes were smaller in LMP2-shRNA group than that in MG132 group although the difference did not reach a significant level (^#^*P*>0.10 compared with MG132 group). Five rats in each group were used for TTC

To examine whether inhibition of LMP2 and MG132 pretreatment reduces infarction in MCAO model, LMP2-shRNAs and Cont-shRNA were designed and cloned into a lentiviral vector to knockdown stroke-induced LMP2 expression and the effects of LMP2 knockdown on infarct volume were then examined *in vivo*. LMP2-ShRNA and MG132 were then injected stereotactically into the right cortex and striatum before MCAO. The transduction of LMP2-shRNA was confirmed by GFP fluorescence and the infarction volume was determined by using 2,3,5-triphenyltetrazolium chloride (TTC) staining. LMP2 knockdown by significantly reduced infarction volumes in ischemic rats. Compared with MCAO+vehicle group (40.8%, infarction volumes/total ipsilateral hemisphere), the infarction volumes were reduced to 26.6% in MCAO+MG132 group (^▴^*P*<0.001, compared with MCAO+vehicle group) (Figure 6A,C). Similarly, the infarction volumes were reduced to 22.5% in LMP2-shRNA group compared with Cont-shRNA group (39.7%, infarction volumes/total ipsilateral hemispheric) (*P<0.001, compared with Cont-shRNA group) (Figure 6B,D). Furthermore, the infarction volumes were smaller in LMP2-shRNA group than that in MG132 group although the difference did not reach a significant level (Figure 6) (^#^*P*>0.10 compared with MG132 group).

### LMP2 knockdown by shRNA reduced activation of astrocytes and microglia after MCAO

**Figure 7:**
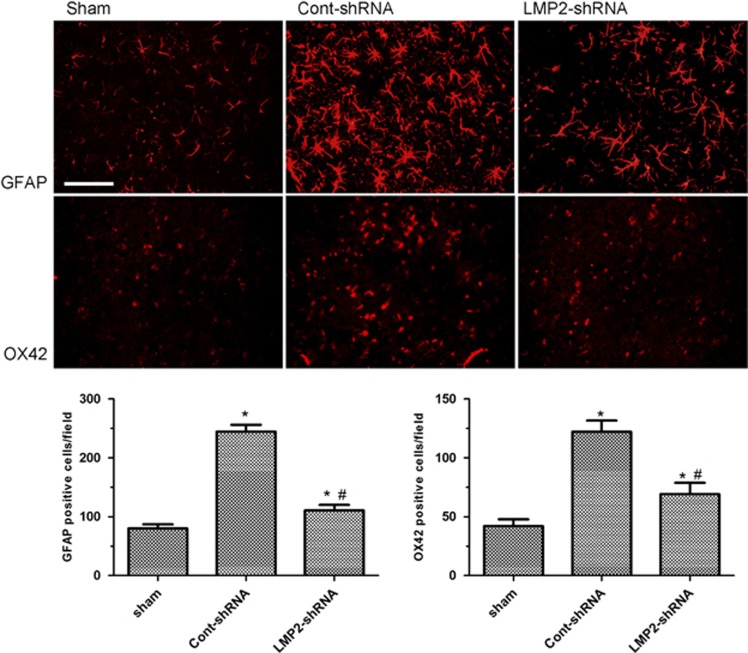
LMP2 knockdown by shRNA reduced activation of astrocytes and microglia after MCAO. Following MCAO, GFAP and OX42 immunoreactivities were evident in the right cortex and striatum in ischemic animals receiving control-ShRNA compared with sham-operated control animals. In contrast, the GFAP and OX42 immunoreactivities were significantly reduced in ischemic animals receiving LMP2-shRNA (*P<0.001, compared with the sham-operated controls; ^#^*P*<0.001 compared with the cont-shRNA group). Scale bars=50 *μ*m. Five rats in each group were used for immunofluorescent assays (*n*=5)

To examine whether inhibition of LMP2 reduces activation of astrocytes and microglia in ischemic rats, LMP2-shRNA was injected stereotactically into the right cortex and striatum before MCAO. There were weak GFAP and OX42 immunoreactivities for astrocytes and microglia in the cortex and striatum in sham-operated control animals without MCAO. Following MCAO, GFAP and OX42 immunoreactivities were evident in the right cortex and striatum in ischemic animals receiving control-shRNA. In contrast, the GFAP and OX42 immunoreactivities were significantly reduced in ischemic animals receiving LMP2-shRNA (*P<0.001, compared with the sham-operated controls; ^#^*P*<0.001 compared with the cont-shRNA group) (Figure 7).

### LMP2 knockdown by shRNA reduced the expression of TNF-*α* and IL-1*β* after MCAO

**Figure 8:**
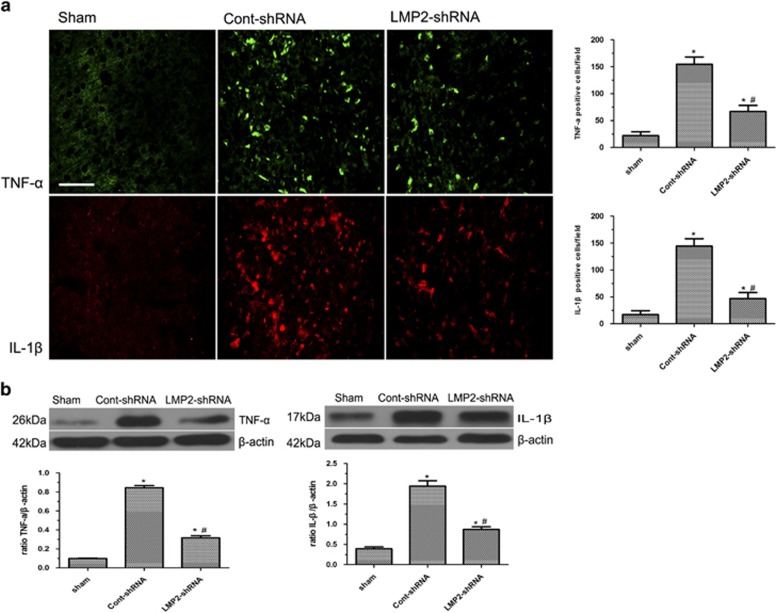
LMP2 knockdown by shRNA reduced the expression of TNF-*α* and IL-1*β* after MCAO. (**a**) Immunofluorescence labeling revealed that both TNF-*α* and IL-1*β* immunoreactivity was significantly upregulated in the ipsilateral ischemic hemisphere compared with sham-operated animals. In contrast, LMP2 knockdown by shRNA significantly reduced the expression of TNF-*α* and IL-1*β* (**A**). Five rats in each group were used for immunofluorescent assays (*n*=5). (**b**) Western blot confirmed that TNF-*α* and IL-1*β* protein levels were reduced to 37.4 and 44.8% compared with the Cont-shRNA group, respectively (*P<0.001, compared with the sham-operated controls; ^#^*P*<0.001 compared with the cont-shRNA group). Scale bars=50 *μ*m. Five rats in each group were used for western blot (*n*=5)

To examine whether inhibition of LMP2 reduces the expression of TNF-*α* and IL-1*β* in ischemic rats, immunofluorescence labeling was used to examine the expression of TNF-*α* and IL-1*β* in ischemic animals receiving either LMP2-shRNA or Cont-shRNA. Immunofluorescence labeling revealed that both TNF-*α* and IL-1*β* immunoreactivity was significantly upregulated in the ipsilateral ischemic hemisphere compared with sham-operated animals (Figure 8A). In contrast, LMP2 knockdown by shRNA significantly reduced the expression of TNF-*α* and IL-1*β*. Western blot confirmed that TNF-*α* and IL-1*β* protein levels were reduced to 37.4 and 44.8% compared with Cont-shRNA group, respectively (*P<0.001, compared with the sham-operated controls; ^#^*P*<0.001 compared with the cont-shRNA group) (Figure 8B).

### Effect of LMP2 knockdown by shRNA and MG132 on the expression of NF-*κ*B and ubiquitinated protein after MCAO

**Figure 9:**
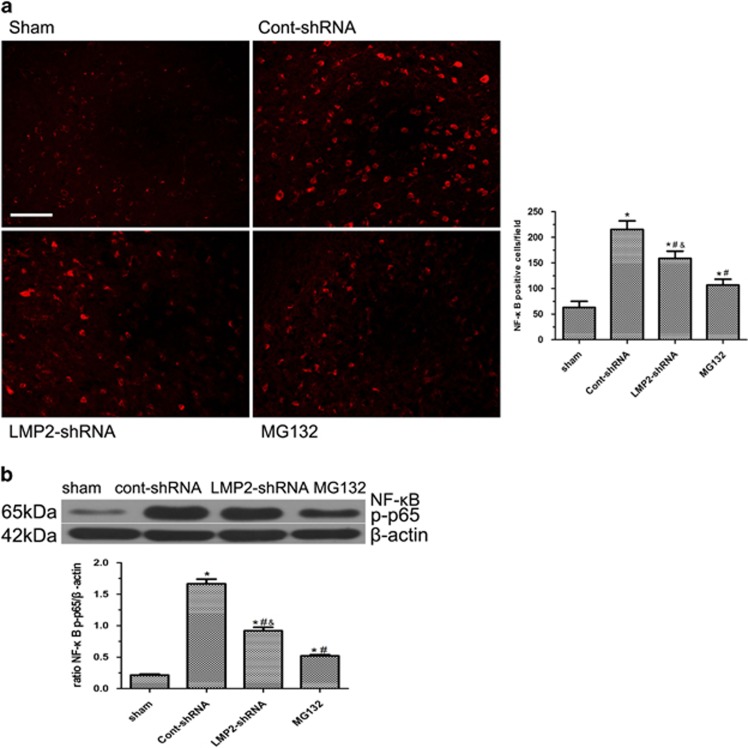
Effect of LMP2 knockdown by shRNA and MG132 on the expression of NF-*κ*B after MCAO. (**a**) The immunofluorescent showed that NF-*κ*B immunoreactivity was significantly increased in the ipsilateral ischemic hemisphere compared with sham-operated animals. In contrast, either LMP2 knockdown by shRNA or MG13 significantly reduced ischemia-induced expression of NF-*κ*B (**A**). Five rats in each group were used for immunofluorescent assays (*n*=5). (**b**) Western blot confirmed that NF-*κ*B p-p65 protein levels was reduced to 55.1 and 31.3% in LMP2-ShRNA and MG132 groups, respectively, when compared with the Cont-shRNA group. However, the NF-*κ*B p-p65 protein levels were lower in MG132 group than that in LMP2-shRNA group (^#^*P*<0.001 compared with the cont-shRNA group; ^&^*P*<0.001 compared with the MG132 group) (**b**). Five rats in each group were used for western blot (*n*=5). Scale bars=50 *μ*m.

To examine the effect of LMP2 knockdown by shRNA and MG132 on the expression of NF-*κ*B in ischemic rats, immunofluorescence labeling was used to examine the expression of NF-*κ*B in ischemic animals receiving either LMP2-shRNA or Cont-shRNA or MG132. The immunofluorescent analysis of rat brain preparations showed that NF-*κ*B immunoreactivity was significantly increased in the ipsilateral ischemic hemisphere compared with sham-operated animals. In contrast, either LMP2 knockdown by shRNA or MG13 significantly reduced ischemia-induced expression of NF-*κ*B (Figure 9A). Western blot confirmed that NF-*κ*B *P*-p65 protein levels was reduced to 55.1 and 31.3% in LMP2-shRNA and MG132 groups, respectively, when compared with the Cont-shRNA group (Figure 9B). Furthermore, the NF-*κ*B *P*-p65 protein levels were lower in MG132 group than that in LMP2-shRNA group (^#^*P*<0.001 compared with the cont-shRNA group; ^&^*P*<0.001 compared with MG132 group) (Figure 9B).

**Figure 10:**
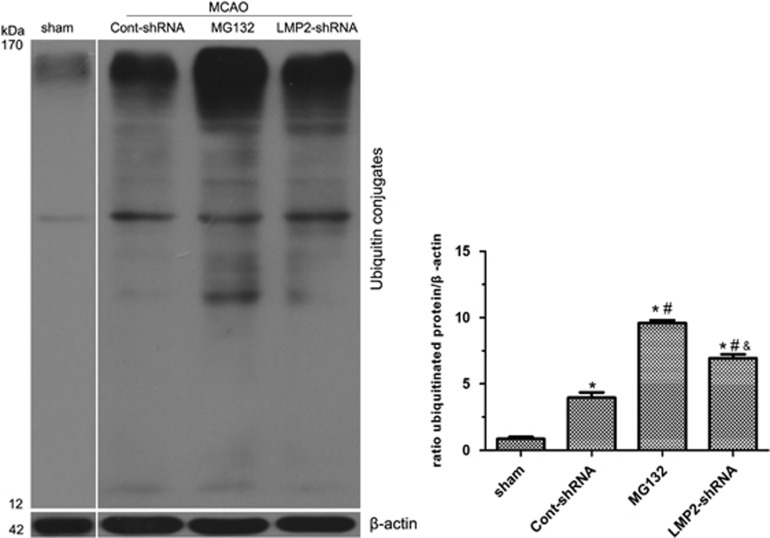
Effect of LMP2 knockdown by shRNA and MG132 on the level of ubiquitinated protein after MCAO. Western blot confirmed that protein ubiquitination was significantly increased after MCAO (*P<0.001, compared with the sham-operated controls). Compared with Cont-shRNA group, protein ubiquitination was increased about 4.2-fold and 1.9-fold in MG132 and LMP2-shRNA groups, respectively (^#^*P*<0.001 compared with the cont-shRNA group; ^&^*P*<0.001 compared with the MG132 group). Five rats in each group were used for western blot (*n*=5)

Protein ubiquitination has been reported in ischemic animals following brain ischemia.^14^ To examine the effects of LMP2 knockdown by shRNA and MG132 on protein ubiquitination in ischemic rats, western blot analysis was used to quantitatively determine the levels of ubiquitinated proteins. Consistently, we found that protein ubiquitination was significantly increased after MCAO (*P<0.001, compared with the sham-operated controls) (Figure 10). Compared with the Cont-shRNA group, protein ubiquitination was increased about 4.2-fold and 1.9-fold in MG132 and LMP2-shRNA groups, respectively (^#^*P*<0.001 compared with the cont-shRNA group; ^&^*P*<0.001 compared with the MG132 group) (Figure 10).

## Discussion

The present study has examined the time course and cellular distribution of immunoproteasome subunit LMP2 and LMP7 protein expression in a rat model of MCAO. We found that LMP2 and LMP7 were robustly induced in inflammatory cells in the ischemic brain as early as 4 h and lasted for 7 days following ischemic stroke. These observations are in line with the process of the brain's inflammatory responses to ischemia, indicating the strong association between immunoproteasome and neuroinflammation under stroke context. Meanwhile, *β*1 and *β*5 standard subunits as well as constitutive proteasome subunits of the 20 S proteasome *α* (1,2,3,5,6,7) did not show a significant change in the same brain region examined under the same conditions. Thus, our findings indicate the presence of subtypes/intermediates type proteasomes in ischemic stroke. More importantly, sh-RNA-mediated inhibition of LMP2 significantly reduced infarction volume and attenuated inflammatory reaction, suggesting that ischemia-induced upregulation of immunoproteasome is detrimental.

Although immunoproteasome is predominantly present in the immune cells, it is also present in non-immune response, suggesting that immunoproteasome may also have non-immune functions.^8, 9, 15^ We found that LMP2 and LMP7 were sparely present in glial-like cells in non-ischemic brain, which is consistent with the notion that the brain is very low in immune activity under physiological condition. In contrast, LMP2 and LMP7 were robustly expressed in immune cells such as astrocytes, microglia and T lymphocytes in ischemic brain. In addition, the expression of LMP2 and LMP7 in ischemic brain was redistributed from cytoplasm to nucleus, indicating that ischemia induced the activation of immunoproteasome. Furthermore, the pattern and time course of LMP2 and LMP7 expression were in line with the expression of inflammatory cytokines such as TNF-*α*, IL-1*β* and NF-*κ*B. Thus, these results are in agreement with the previous studies that immunoproteasome is highly expressed in immune cells in brain diseases.^11, 16^ Interestingly, although immunoproteasome activity has been detected in different nervous tissue cell types, the major cell types expressing immunoproteasome vary in different neurological diseases. For example, neurons have been reported to be a major source of LMP2 and LMP7 in HD model. On the other hand, LMP2 and LMP7 have been detected mainly in immune cells in an amyotrophic lateral sclerosis model. Furthermore, immunoproteasome is exclusively expressed in immune cells to exacerbate immunopathological damage in a model of meningitis, suggesting a critical role of immune cells in immunoproteasome-associated injury in the inflamed brain.^16^ Several possibilities have proposed to explain the discrepancy such as brain region-specific and pathological differences in the proteasome induction response. Our results are in contrast to those of a previous study suggesting that neurons are the major sources of LMP2 and LMP7 in an ischemic stroke model. In that study, LMP2 and LMP7 were detected in a mouse model of mild transient ischemia (30 min).^13^ Although immunity and inflammation are involved in all stages of the ischemic cascade, the magnitude of inflammatory responses is associated with severity of ischemia. Thus, a prolonged ischemic model as used in the present study may be required to induce the immunoproteasome-associated inflammatory responses.^1^

Immunoproteasome has been reported to have either a beneficial or detrimental role depending on the experimental context.^8, 10, 16, 17, 18^ In the present study, we further addressed whether immunoproteasome represents a pro-survival or a deleterious event under stroke context. The immunoproteasome has three specific catalytic subunits including LMP2, MECL-1 and LMP7. It is generally believed that LMP2 is a critical component for proteasome activity in that LMP2 is essential for the proper assembly of the immunoproteasome.^19^ However, recent studies have shown that there are different subtypes/intermediate type proteasomes in several tissue and organs where the LMP7 subunit can be incorporated along with standard catalytic subunits.^20, 21^ Thus, it is necessary to test the role of LMP2 as a regulator for i-proteasomes assembly in cerebral ischemia. We further examined the effects of sh-RNA-mediated knockdown of LMP2 on infarct volume. Animals with shRNA-mediated LMP2 knockdown exhibited a significant small infarct volume compared with control animals, indicating a potent detrimental role of LMP2 in ischemic stroke. Furthermore, shRNA-mediated knockdown of LMP2 also significantly reduced ischemia-activated immune cells including microglia and astrocytes. Immunoproteasome has been reported to exert its inflammatory action by regulation of activation of NF-*κ*B. It is not clear whether NF-*κ*B is activated by immunoproteasome during cerebral ischemia. To address this question, we examined the effects of LMP2 knockdown on the level of NF-*κ*B after ischemia. We found that ischemia dramatically increased NF-*κ*B P65 expression whereas LMP2 knockdown significantly attenuated ischemia-induced NF-*κ*B P65 level. Similarly, LMP2 knockdown also significantly reduced TNF-*α* and IL-1*β* level in ischemic animals. Given that TNF-*α* and IL-1*β* are the downstream genes of NF-*κ*B, these results suggest that the LMP2 may mediate ischemic damage through regulation of NF-*κ*B.

The clinical uses of general proteasome inhibitors are often limited by their side effects due to their non-selective inhibition of protein degradation.^6^ To address whether selective inhibition of LMP2 immunoproteasome reduces side effects, we examined the effects of shRNA-mediated knockdown of LMP2 on proteolytic activities and polyubiquitylated proteins. Ischemia induced a robust increase in C-L and CT-L activities whereas LMP2 knockdown caused nearly 6-fold decrease in C-L and CT-L activities compared with the control group. Consequently, polyubiquitylated proteins were significantly accumulated in animals with LMP2 knockdown. Interestingly, LMP2 knockdown caused much less accumulation of polyubiquitylated proteins compared with general proteasome inhibitor MG132 treatment although both LMP2 knockdown and MG132 pretreatment achieved similar reduction of infarction volumes in ischemic rats. Collectively, these results suggest that LMP2 knockdown protects against ischemic insult without severely disturbing overall proteasome function. However, the present study has several limitations. For example, we could not completely rule out the possible role of non-inflammatory functions of immunoproteasome in ischemic stroke. Thus, further studies are required to understand fully the mechanism by which immunoproteasome exacerbates ischemic injury. In addition, MG132 is a non-selective proteasome inhibitor. Thus, MG132-induced protection may be in part related to inhibition of standard proteasomes. Future study using specific immunoproteasome inhibitors is warranted to explore the therapeutic potential of specific immunoproteasome inhibitors in cerebral ischemia. Nevertheless, the present data suggest that augmentation of immunoproteasome may be involved in regulating inflammation cytokines production via regulating activation of NF-*κ*B in ischemia stroke whereas selective inhibition of the immunoproteasome subunit affords a strong reduction of infarction volumes and a suppression of proinflammatory cytokines after MCAO, indicating that immunoproteasome-specific inhibitor might be an effective anti-inflammation agent for stroke treatment.

In conclusion, the present study demonstrates that both LMP2 and LMP7 are mainly produced from inflammatory cells and these two immunoproteasome units also undergo time-dependent changes after cerebral ischemia-reperfusion. In addition, augmentation of immunoproteasome may be potentially involved in inflammatory pathophysiological mechanism of ischemia stroke. We also infirm that selective inhibition of the immunoproteasome subunit offers a strong reduction of infarction volumes and a suppression of proinflammatory cytokines after MCAO. We postulate that interfering with immunoproteasome with highly selective proteasome inhibitor may be a new paradigm for acute treatment of stroke, as well as other neurological diseases.

## Materials and Methods

### Ethical approval

All experiments were approved by the Institutional Animal Ethical Committee of Sun Yat-sen University (the Guide for the Care and Use of Laboratory Animal of the National Institute of Health, Publication No. 80-23, revised 1996) and performed to comply with the ARRIVE (Animal Research: Reporting In Vivo Experiments) guidelines. Male Sprague–Dawley rats (10 weeks old) weighing 250–300 g were purchased from the Center for Experimental Animals of Sun Yat-Sen University. Animals were maintained in a temperature controlled room, on a 12/12-h light/dark cycle with food and water *ad libitum*. They were housed five per cage to maintain social interaction. To reduce the animal's stress level, the same operator performed all steps of experiments. Efforts were made to reduce the number of rats. A total of 126 rats were used in the completion of this study, and finally only 90 rats were involved in this experiment. Rats were randomly assigned into six groups: sham-operated control group (sham, *n*=10), MCAO group (MCAO, *n*=40), MCAO+lentivirus-mediated LMP2 shRNA (referred to as LMP2-shRNA) (LMP2-shRNA, *n*=10), MCAO+lentivirus vector carrying scrambled shRNA (referred to as Cont-shRNA) (Cont-shRNA, *n*=10), MCAO+MG132 group (MG132, *n*=10) and MCAO+vehicle group (vehicle, *n*=10).

### MCAO model

Rats were anesthetized with intraperitoneal (IP) injection of 10% chloral hydrate (3 ml/kg body weight) and subjected to MCAO as described previously, with minor modifications.^22^ In brief, a midline neck incision was made, and the right common carotid artery (CCA), external carotid artery (ECA) and internal carotid artery (ICA) were isolated. The ECA was tied. A 4-0 monofilament nylon suture (Beijing Sunbio Biotech Co. Ltd, Beijing, China) with a rounded tip was aseptically inserted from the right CCA to the ICA through the stump of the ECA and gently advanced to occlude the MCA. Recirculation/reperfusion of cerebral blood flow was allowed by gently removing the monofilament after 1-h ischemia, followed by different time intervals of reperfusion. In sham-operated animals, all procedures except occlusion of the MCA were performed. Core body temperatures were monitored with a rectal probe and maintained at 37 °C during the whole procedure. Following surgery, rats were allowed to recover spontaneous breathing and were kept in their cages with free access to food and water. To evaluate impairment of neuronal function after stroke, neurologic examinations were performed 2, 4 and 8 h after the onset of occlusion and then daily until killing by a blinded examiner who used a modified scoring system based on that developed by Longa *et al.*^22^ The scoring system used is as follows: 0, no deficits; 1, difficulty in fully extending the contralateral forelimb; 2, unable to extend the contralateral forelimb; 3, circling to the contralateral side; 4, falling to the contralateral side; 5, did not walk spontaneously and displayed a depressed level of consciousness.

### Lentiviral construction preparation and injection

Four shRNA sequences targeting rat LMP2 (GenBank, Psmb9, NM_012708) and a negative control sequence were constructed by Genechem (Shanghai, China). The best-performing LMP2 shRNA sequence was CCTGGTCACCATTACAGCT, and the negative control scrambled shRNA sequence was CCATCATGGCTGTGGAATT. ShRNAs were inserted into a hU6-MCS-CMV-EGFP lentivirus vector containing a CMV-driven EGFP reporter gene and a U6 promoter upstream of restriction sites (*Hpa*I and *Xho*I) (Genechem, China). All constructs were confirmed by sequence analysis. Recombinant lentiviruses were produced by co-transfecting 293T cells with the aid of Lipofectamine 2000 (Invitrogen, Irvine, CA, USA, 11668-500) according to standard protocols. Virus titers, expressed as transducing units (TU) per milliliter, were determined by measuring GFP expression in 293T cells, which were transduced with serial dilutions of concentrated lentivirus. The titers were approximately 4 × 10^9^ TU/ml. Lentivirus preparations were infused stereotactically into the ipsilateral hemispheric region 1 h before MCAO. Briefly, rats were anesthetized as above and placed on a stereotactic apparatus. ShRNA preparations were delivered into the right ishcemia region using a 15-ml syringe at the following coordinates (10 *μ*l/per site): bregma backward 1 mm, 15 mm lateral, 4 mm dorsoventral. The rats were allowed to recover for up to 3 days to enable sufficient gene expression.

### Detection of fluorescence labeled LMP2-shRNA

At 72 h after injection, rats were killed under deep anesthesia with 10% chloral hydrate (5 ml/kg body weight, IP) and then transcardially perfused with 0.9% sodium chloride at 4 °C followed by 4% paraformaldehyde in 0.01 M phosphate-buffered saline (PBS, pH 7.4). Brains were removed, shock frozen in liquid nitrogen, and cryosections were prepared. Fluorescence was analysed using a fluorescence microscope (BX51; Olympus, Tokyo, Japan).

### Drug administration

MG132 (dissolved in 2% dimethyl sulphoxide in saline; Sigma, St Louis, MO, USA) and vehicle (2% dimethyl sulphoxide in saline) were stereotactically injected into the ipsilateral hemispheric region 1 h before MCAO. In brief, rats were anesthetized as above and mounted on a stereotaxic apparatus and fixed accordingly. The skull was exposed and a hole was drilled in the skull overlying the right hemisphere at the following coordinates: bregma backward 1 mm, 15 mm lateral and 4 mm dorsoventral. In all, 10 *μ*l of either MG132 (2 mg/kg body weight) or 2% dimethyl sulphoxide in saline (vehicle control animals) was delivered into the right ishcemia region using a 15-ml syringe. The rats were allowed to recover for up to 3 days to enable sufficient gene expression. Importantly, the doses of MG132 used in the present study were chosen from previous experiments.

### Tissue preparation

At 4, 24, 72 h and 7 days after reperfusion, five rats from each group were killed under deep anesthesia with 10% chloral hydrate (5 ml/kg body weight, IP) and then transcardially perfused with 0.9% sodium chloride at 4 °C followed by 4% paraformaldehyde in 0.01 M PBS (pH 7.4). Brains were then removed, kept in the same fixative for 48 h at 4 °C and cryoprotected in serial PBS isopropanol sucrose solutions (20 and 30%) at 4 °C until brains sank. Coronal sections (10 *μ*m) were cut on a cryostat (CM1900; Leica, Heidelberger, Germany) and used for immunofluorescent staining.

### Measurement of cerebral infarction volume

The measurement of cerebral infarct volume was performed as previously described.^5, 23, 24^ From each rat brain, analysis of ischemic damage included core infarct volume from both cortical and subcortical brain regions. The animals were euthanized 72 h after reperfusion by chloral hydrate (350 mg/kg, IP) anesthesia overdose. The brains were rapidly dissected out and the forebrains were cut into six coronal sections (2 mm thick). The sections were stained by incubating them in a solution of 2% TTC (Sigma-Aldrich, St Louis, MO, USA) at 37 °C for 20 min. Then, the infarcted brain tissue appeared white, whereas the non-infarcted region appeared red. The sections were fixed with 4% paraformaldehyde and then digitized. The cross-sectional areas with or without infarction in each brain slice were measured using Image J analysis software (ImageJ 1.6q software, U.S. National Institutes of Health, Bethesda, MA, USA). Infarct volume was determined by integrating the crosssectional area of infarction at each stereotaxic level and the thickness of slice according to the following formula: *V*=*t* × (*A*1+*A*2+…*An*). *V* is the infarct volume (mm^3^), *t* is the thickness of slice and *A* is the infarct area (mm^2^). Correction for edema of infarct area was performed as described by Lin *et al.*^25^

### Immunofluorescent labeling

For immunofluorescent assays, frozen sections (10 *μ*m) were prepared using a cryostat (Leica, CM1900) according to standard procedures. The following antibodies were used: rabbit anti-LMP2 (1 : 400; Abcam, Cambridge, UK), rabbit anti-LMP7 (1 : 300; Abcam), mouse anti-NeuN (1 : 400; Chemicon, Temecula, CA, USA), mouse anti-rat GFAP (1 : 800; Cell Signaling Technology, Beverly, MA, USA), mouse anti-rat OX-42 (1 : 300; Millipore, Billerica, MA, USA), mouse anti-CD31 (1 : 100; AbD Serotec, Kidlington, UK), mouse anti-CD4 (1 : 100; AbD Serotec), goat anti-rat-TNF-a (1 : 1000; R&D systems, Minneapolis, MN, USA), goat anti-rat-IL-1*β* (1 : 1000; R&D Systems). Immunofluorescence was performed as described previously.^26, 27^ Briefly, sections were pre-incubated with 0.3% Triton X-100 (v/v) in 0.01 M PBS (pH 7.4) for 10 min, followed by blocking in 10% normal goat serum (Kirkegaard & Perry Laboratories, Inc., Maryland, WA, USA) or 1% bovine serum albumin (MPBIO, Santa Ana, CA, USA) for 1 h at room temperature. Sections were then incubated overnight at 4 °C with primary antibodies diluted in primary antibody diluents (DAKO, Glostrup, Denmark). After rinsing in 0.01 M PBS (3 × 5 min), sections were incubated with FITC-goat anti-rabbit IgG antibodies (1 : 250; Kirkegaard & Perry Laboratories) or Alexa Fluor 555 conjugated goat anti-rabbit IgG (H+L), F(ab')2 Fragment (1 : 1000; Cell Signaling Technology, Danvers, MA, USA) or Alexa Fluor 555 conjugated goat anti-mouse IgG (H+L), F(ab')2 Fragment (1 : 1000; Cell Signaling Technology) in 0.01 M PBS for 1 h at room temperature. Finally, sections were thoroughly washed (3 × 5 minutes). If necessary, sections were counterstained for nuclei with 4′,6-diamidino-2-phenylindole dihydrochloride (DAPI; 1 : 1000; Roche, Mannheim, Germany), and then mounted in ProLong Gold antifade reagent (Invitrogen) before imaging. Fluorescence signal was detected with a microscope (BX51; Olympus). Negative control sections were incubated with PBS instead of primary antibodies and showed no positive staining. Five rats in each group were used for immunofluorescent assays (*n*=5).

### Western blot analyses

Rats were killed as above and then transcardially perfused with 0.9% sodium chloride at 4 °C. Brains were then removed, the ipsilateral ischemic cortex and striatum around the infarct area was rapidly dissected from the brain tissue and then homogenized in cell lysis buffer (Thermo Scientific Pierce, Rockford, IL, USA) with complete protease inhibitor cocktail (Roche). Brain lysates were centrifuged (16 400 rpm for 30 min), and then protein concentrations were determined using a Bicinchoninic Acid (BCA) Protein Assay kit (Thermo Scientific Pierce) according to the manufacturer's instructions. Soluble protein (50 *μ*g) was separated by 4–20% gradient SDS/PAGE (sodium dodecyl sulfate polyacrylamide gel electrophoresis) (Bio-Rad Laboratories Inc, Hercules, CA, USA) and then transferred onto polyvinylidene fluoride membrane (Millipore). Membranes were blocked with Tris-buffered saline containing 0.1% Tween-20 (TBST) and 5% non-fat milk (MERBCON, Bedford, MA, USA, BCR685). The membranes were then incubated with primary antibodies. Primary antibodies used were as follows: rabbit anti-LMP2 (1 : 2000; Abcam), rabbit anti-LMP7 (1 : 1000; Abcam), mouse anti-20 S alpha subunits (1,2,3,5,6,7) (1 : 500; Abcam), rabbit anti-20 S proteasome *β*1, rabbit anti-20 S proteasome *β*5 (1 : 200; Santa Cruz Biotechnology, Inc., Santa Cruz, CA, USA), rat anti-interleukin 1 beta (IL-1ß) (1 : 1000; Abcam), Phospho-NF-*κ*B p65 (Ser536) (93H1) rabbit monoclonal antibody (1 : 1000; Cell Signaling Technology), goat anti-tumor necrosis factor alpha (TNF-*α*) (1 : 5000; Novus Biologicals, Littleton, CO, USA), mouse monoclonal anti-*β*-actin (1 : 3000; Proteintech Group Inc., Chicago, IL, USA) served as the housekeeping protein. Membranes were exposed to the secondary antibodies diluted in blocking buffer for 1 h at room temperature: horseradish peroxidase (HRP)-conjugated goat anti-mouse (1 : 6000; EarthOx Life Sciences, San Francisco, CA, USA), HRP-conjugated goat anti-rabbit (1 : 3000; Cell Signaling Technology), or HRP-conjugated rabbit anti-goat IgG antibodies (1 : 3000; Invitrogen). Immunoreactivity was detected with Chemiluminescent HRP Substrate (Millipore) for 5 min and then exposed to Kodak X-OMAT films. The exposed X-ray films were scanned. The optical densities were normalized to those of *β*-actin and calculated as target protein expression/*β*-actin expression ratios (ImageJ 1.42q software, U.S. National Institutes of Health). Five rats in each group were used for western blot (*n*=5).

### Measurement of 20S proteasome activity in brain lysates

The remaining portions of the ipsilateral ischemic cortex and striatum around the infarct area samples (≈50 mg) were individually homogenized in cell lysis buffer (Thermo Scientific Pierce) by crushing with mortar and pestle on ice, then dispersed with a sonicator (Kontes micro-ultrasonic cell disrupter). Samples were centrifuged (16 400 rpm for 30 min), and then protein concentrations were determined by using a bicinchoninic acid (BCA) protein assay kit (Thermo Scientific Pierce) according to the manufacturer's instructions before storage at −80 °C. Proteasome activity assays were performed as previously reported,^28, 29, 30^ using the Proteasome Activity Assay Kit (Chemicon, Billerica, MA, USA). The detection of proteolytic activity is based on the release of free 7-amino-4-methylcoumarin (AMC) fluorophore. In brief, to determine chymotrypsin-and caspase-like activity, 150 and 200 *μ*g of protein, respectively, was incubated in assay buffer including 250 mmol/L HEPES HCl, pH 7.5, 5 mmol/L EDTA, 0.5% Nonidet-P40, and 0.1% sodium dodecyl sulfate (SDS) (w/v), with 50 *μ*mol/L substrate (Suc- LLVY-AMC for Ch-T-L activity and Boc-LAA-AMC for T-L activity) in a final volume of 100 *μ*l in 96-well plates for 1 h at 37 °C according to the manufacturer's instructions (Chemicon). Fluorescence of substrate cleavage was determined at 37 °C in a spectrofluorometer (Thermo Electron, Waltham, MA, USA) at excitation and emission wavelengths of 380 and 460 nm, respectively. Proteasome activities for samples were expressed as relative fluorescence units/min/*μ*g brain tissue. Several controls were performed in parallel with measurement of proteasome activity, including standard curves using AMC, protein-free blanks and substrate-only measures. The analyses were conducted in the presence of a specific inhibitor of the proteasome lactacystin (50–200 *μ*mol/L) to ensure the specificity of the proteasome assays.^28, 30, 31, 32^ Five rats in each group were used for measurement of 20S proteasome activity (*n*=5).

### Image analysis and quantification

All histological images were analyzed with Image-Pro Plus image analysis software (Media Cybernetics, Silver Spring, MD, USA) by one blinded assessor. The number of immunostaining positive cells was counted using Image-Pro Plus image analysis software in nine comparable, non-overlapping fields (425 × 320 *μ*m; 3 fields per section × 3 sections per rat) under × 400 magnification and was presented as the average cell number per field on each section.^26, 27^

### Statistical analysis

Results were expressed by means±s.d. Statistical analysis was performed by one-way analysis of variance followed by LSD test for *post hoc* analysis. Differences were considered as significant when *P*<0.05.

## Abbreviations

LMP2: low-molecular-weight protein 2
LMP7: low-molecular-weight protein7
MECL-1: multicatalyticendopeptidase complex-like-1
TNF-α: tumor necrosis factor-α
IL-1β: interleukin-1β
NF-κB: nuclear factor-κB
HD: Huntington's disease
AD: Alzheimer's disease
CNS: central nervous system
GFAP: glial fibrillary acid protein
IP: ipsilateral hemisphere
ctrl: contralateral hemisphere
sham: sham-operated control rats
ARRIVE: Animal Research: Reporting In Vivo Experiments
MCAO: middle cerebral artery occlusion
CCA: common carotid artery
ECA: external carotid artery
ICA: internal carotid artery
shRNA: short hairpin RNA
C-L: caspase-like
CT-L: chymotrypsin-like
PBS: phosphate-buffered saline
DAPI: 4′,6-diamidino-2-phenylindole dihydrochloride
TTC: 2,3,5-triphenyltetrazolium chloride
AMC: 7-amino-4-methylcoumarin
BCA: bicinchoninic acid
